# Supplementary material for: Transcription Factor Homeobox D9 Drives the Malignant Phenotype of HPV18-Positive Cervical Cancer Cells via Binding to the Viral Early Promoter
Source: Cancers (Basel). 2021 Sep 15;13(18):4613. doi: 10.3390/cancers13184613 (PMC8470817; doi:10.3390/cancers13184613)
Supplement: Supplementary file 1 [file cancers-13-04613-s001.zip › cancers-1328127-Table S2 and Figure S1.pdf]

Supplementary Materials: Transcription Factor Homeobox D9 Drives the Malignant Phenotype of HPV18-Positive Cervical Cancer Cells via Binding to the Viral Early Promoter

Shigenori Hayashi, Takashi Iwata, Ryotaro Imagawa, Masaki Sugawara, Guanliang Chen, Satoko Tanimoto, Yo Sugawara, Ikumo Tanaka, Tomoya Matsui, Hiroshi Nishio, Masaru Nakamura, Yuki Katoh, Seiichiro Mori, Iwao Kukimoto and Daisuke Aoki

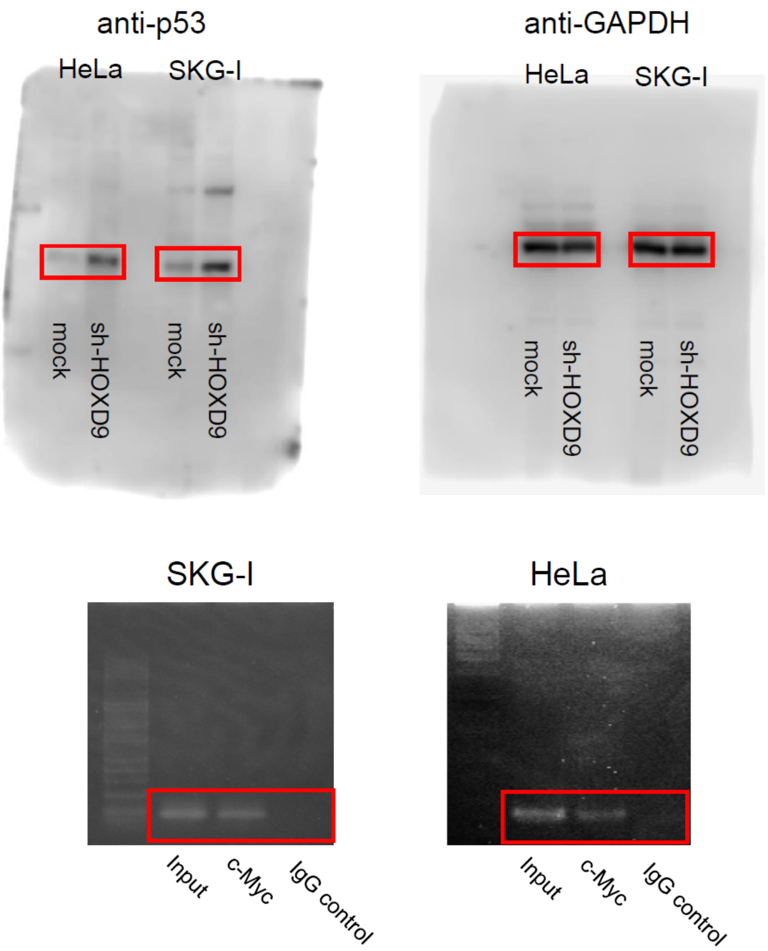

Figure S1: Original western blot figures for Figure 2D and 4B.

Table S2. The top 20 inhibited upstream regulators predicted by IPA.

Upstream Regulator	Molecule Type	Activation z-Score	p-Value of Overlap
IFNA	cytokine	-5.742	0.0000302
IRF7	transcription regulator	-4.990	0.00142
IFNL1	cytokine	-4.949	0.0000141
IKBKG	kinase	-4.176	0.0408
poly rI:rC-RNA	biologic drug	-4.074	0.0000491
Ifn	group	-4.036	0.0233
TBX2	transcription regulator	-4.031	0.0000553
DDX58	enzyme	-3.926	0.000599
RABL6	other	-3.922	0.000169
CHUK	kinase	-3.766	0.000195
TLR3	transmembrane receptor	-3.761	0.00341
E2f	group	-3.568	0.000703
Ifnar	group	-3.530	0.0638
PDGF BB	complex	-3.518	0.0000718
IRF5	transcription regulator	-3.475	0.0413
IKBKB	kinase	-3.414	0.0114
CCND1	transcription regulator	-3.270	0.000379
TBK1	kinase	-3.186	0.00818
SREBF2	transcription regulator	−3.000	0.0259
stallimycin	biologic drug	−2.917	0.0022
